# Comparative genomics of first available bovine *Anaplasma phagocytophilum* genome obtained with targeted sequence capture

**DOI:** 10.1186/1471-2164-15-973

**Published:** 2014-11-17

**Authors:** Thibaud Dugat, Valentin Loux, Sylvain Marthey, Marco Moroldo, Anne-Claire Lagrée, Henri-Jean Boulouis, Nadia Haddad, Renaud Maillard

**Affiliations:** Université Paris-Est, Ecole Nationale Vétérinaire d’Alfort, UMR BIPAR ENVA Anses UPEC USC INRA, Maisons-Alfort, France; INRA, UR1077 Mathématique, Informatique et Génome, Jouy-En-Josas, France; Centre de Ressources Biologiques Génomique des Animaux Domestiques et d’Intérêt Economique, INRA, Jouy-En-Josas, France; Unité pathologie des ruminants, Ecole Nationale Vétérinaire de Toulouse, Toulouse, France

**Keywords:** *Anaplasma phagocytophilum*, Cattle, Comparative genomics, Granulocytic anaplasmosis, Tick-borne fever, Targeted sequence capture, Whole genome sequencing

## Abstract

**Background:**

*Anaplasma phagocytophilum* is a zoonotic and obligate intracellular bacterium transmitted by ticks. In domestic ruminants, it is the causative agent of tick-borne fever, which causes significant economic losses in Europe. As *A. phagocytophilum* is difficult to isolate and cultivate, only nine genome sequences have been published to date, none of which originate from a bovine strain.

Our goals were to; 1/ develop a sequencing methodology which efficiently circumvents the difficulties associated with *A. phagocytophilum* isolation and culture; 2/ describe the first genome of a bovine strain; and 3/ compare it with available genomes, in order to both explore key genomic features at the species level, and to identify candidate genes that could be specific to bovine strains.

**Results:**

DNA was extracted from a bovine blood sample infected by *A. phagocytophilum*. Following a whole genome capture approach, *A. phagocytophilum* DNA was enriched 197-fold in the sample and then sequenced using Illumina technology. In total, 58.9% of obtained reads corresponded to the *A. phagocytophilum* genome, covering 85.3% of the HZ genome. Then by performing comparisons with nine previously-sequenced *A. phagocytophilum* genomes, we determined the core genome of these ten strains. Following analysis, 1281 coding DNA sequences, including 1001 complete sequences, were detected in the *A. phagocytophilum* bovine genome, of which four appeared to be unique to the bovine isolate. These four coding DNA sequences coded for "hypothetical proteins of unknown function” and require further analysis. We also identified nine proteins common to both European domestic ruminants tested.

**Conclusion:**

Using a whole genome capture approach, we have sequenced the first *A. phagocytophilum* genome isolated from a cow. To the best of our knowledge, this is the first time that this method has been used to selectively enrich pathogenic bacterial DNA from samples also containing host DNA. The four proteins unique to the *A. phagocytophilum* bovine genome could be involved in host tropism, therefore their functions need to be explored.

**Electronic supplementary material:**

The online version of this article (doi:10.1186/1471-2164-15-973) contains supplementary material, which is available to authorized users.

## Background

The dramatically reducing cost of high-throughput sequencing (HTS) technologies has enabled their use across a wide range of bacterial genome sequencing projects [1, 2]. HTS can now even be used for routine medical investigations in bacteriology [3, 4]. To effectively perform whole genome sequencing (WGS) on obligate intracellular bacteria, microorganism isolation, culture and DNA purification are often essential steps. The difficulty, or even impossibility, of cultivating some of these bacteria, can be a critical barrier to accessing their genomic sequences. Some authors attempted to sequence the genomes of intracellular bacteria without culturing steps, but successful approaches seem to be rare [5, 6]. Such difficulties result in an underutilization of HTS technologies when studying this type of bacteria.

*Anaplasma phagocytophilum* is a tick-borne alpha-proteobacterium [7]. It infects a large range of hosts, including humans, wild and domestic ruminants, dogs, horses, and rodents [8]. This bacterium is challenging to isolate and cultivate, as it replicates in short lifespan cells (*i.e.* polynuclear cells), which rapidly undergo autolysis after sampling. For this reason, only nine *A. phagocytophilum* genomes are currently available, of which just three are complete [9, 10]. Apart from Norway Variant 2, obtained from a Norwegian sheep, all genomes correspond to North American strains: human strains HZ, HZ2, and HGE1, Dog2 dog strain, MRK horse strain, JM rodent strain, and the tick (*Ixodes scapularis*) strains CRT38 and CRT35. *A. phagocytophilum* is the causative agent of granulocytic anaplasmosis in humans, horses, dogs and occasionally cats, and tick-borne fever (TBF) in domestic ruminants [8] *.* However, the epidemiology of *A. phagocytophilum* infection differs greatly between Europe and the USA. In the USA, Human granulocytic anaplasmosis (HGA) is an increasing public health problem, with a five-fold increase in the number of cases between 2000 and 2010 [11], whereas no TBF cases have been described to date. In contrast, HGA appears to be rare in Europe (however the number of reported cases has increased during recent years, probably related, at least in part, to improved diagnostic tools and surveillance [12, 13]), whereas TBF cases are severe in cattle and sheep, causing significant economic losses [14–16].

These different epidemiological contexts are associated with considerable strain variations [17, 18]. An American strain infectious for horses is not infectious for ruminants [19], while a European variant pathogenic for cattle does not cause any clinical disease in horses [20]. In the USA, the Ap-Variant 1 infects goats and deer, but not mice [21–23], whereas the Ap-ha variant can infect both ruminants and mice under experimental conditions [21, 22, 24]. Taken together, these results suggest that distinct *A. phagocytophilum* ecotypes with varying host tropisms, circulate in Europe and the USA. The genetic diversity of *A. phagocytophilum* must be explored in order to investigate its phylogeny, and to also identify genetic markers capable of distinguishing ecotypes. For this purpose, genome sequences from various animal strains are needed. Additionally, given the significant economic consequences of TBF infection in Europe, particularly in France, it is important to specifically focus on bovine *A. phagocytophilum*.

Within this context, our objective was to sequence and characterize the genome of *A. phagocytophilum* (hereafter referred to as ‘BOV-10_179’) obtained from a cow (*Bos taurus*) with TBF, and generate comparisons with the other available *A. phagocytophilum* genomes. Therefore we followed a whole genome solid-phase sequence capture approach, which allowed us to sequence the genome of a cow sample without the need for strain isolation. This strategy has already been successfully used to sequence the genome of the arthropod symbiotic bacterium *Wolbachia*, another obligate intracellular microorganism [5, 25]. To the best of our knowledge, our study is the first to provide the genome sequence of a bovine strain. Following comparison with nine available genomes, we then identified core and accessory *A. phagocytophilum* genes. Core genes comprised the set of orthologous genes shared among the ten sequenced *A. phagocytophilum* genomes, whereas accessory genes were those orthologous genes not shared among the ten genomes. Four genes were specific to the *A. phagocytophilum* bovine genome, and nine were common to both genomes from domestic ruminants (*ie*. a cow and a sheep). As all of these genes code for “proteins of unknown function without similarity to other proteins” their functions must now be explored.

## Results and discussion

### Confirmation of *A. phagocytophilum*infection

**Figure 1:**
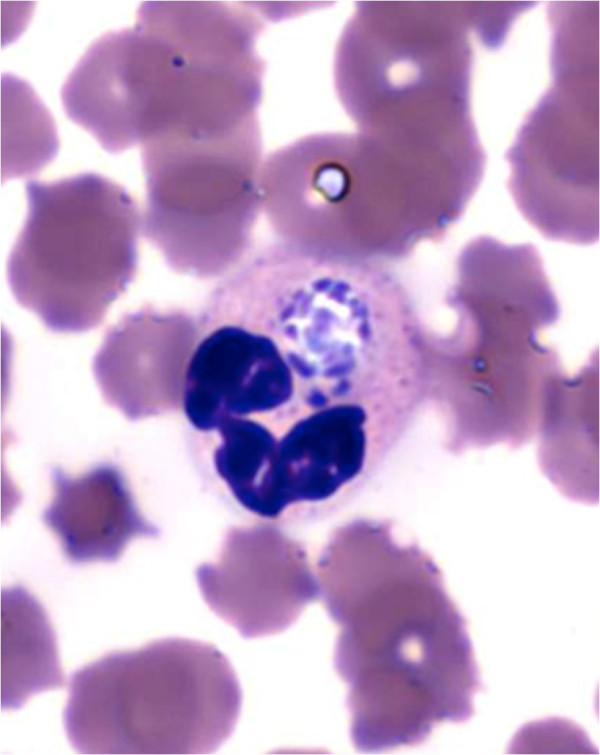
**Morulas observed in the neutrophils of the bovine blood sample 10_179.**

In February 2010, one cow (10_179) was diagnosed with TBF at the National Veterinary School of Toulouse (France). *A. phagocytophilum* infection was confirmed by the observation of morulas in blood smears (Figure 1) and by *msp2* PCR amplification. Another cow sample (bovine 1), was confirmed as uninfected by *A. phagocytophilum* (absence of both morulas and *msp2* amplification), and was used as a negative control.

### Whole genome capture and sequencing of *A. phagocytophilum*

The whole genome capture step was performed using a NimbleGen solid-phase capture array which encompassed 1,458,085 bp, corresponding to 99.1% of the HZ reference strain genome. The library thus obtained was sequenced using the Illumina HiSeq 2000 platform with a paired-end protocol, and a total of 379,038,930 reads were retrieved. Subsequently, these reads were aligned to *A. phagocytophilum* and cattle genomes. A total of 223,159,632 properly paired reads mapped to the pathogen genome, corresponding to 58.9% of the total number of reads. This percentage is similar to that obtained by other groups, such as by Bright *et al.* [26]. On the other hand, 24,532,400 properly paired reads (6.5%) were unmapped.

After the alignment step, the reads mapping to the cattle genome were discarded and not considered for subsequent analysis. For the subset of reads which mapped to the pathogen genome, further PCR duplicate detection was performed. PCR-duplicated reads are known to arise during the final amplification steps of library preparation, and are related to the reduced complexity of captured libraries. In our case, 148,695,448 duplicated reads were found in the dataset mapping to *A. phagocytophilum*, which corresponded to a percentage of 66.6%. This value is relatively high as compared to those obtained by other authors in similar contexts. For instance, Bright et *al.* (2012) reported to have obtained percentages of duplicated reads ranging from 12.1% to 46.2% in *Plasmodium vivax* field samples, with an average of 26.1% [26].

The properly paired *A. phagocytophilum* reads retained after the removal of duplicates (74,464,184) were combined with the properly paired unmapped reads (24,532,400, as previously stated) to obtain a final dataset of 98,996,584 reads, roughly corresponding to 9.9 Gb of data and representing a sequencing depth of 6,728X. Due to the massive amount of raw data, the reads were first digitally normalized using Khmer [27, 28]. Subsequently, 14,791,364 properly paired reads were retained (about 986X of coverage) and then *de novo* assembled using Velvet. The assembly resulted in 169 scaffolds (199 contigs) with lengths of 1 kb or more. The N50 value was 14,519 bp with a maximum length of 59.6 kb and a minimum length of 1,009 bp. The total length of the assembly was 1,370,818 nucleotides, consistent with the sizes of other *A. phagocytophilum* genomes.

**Figure 2:**
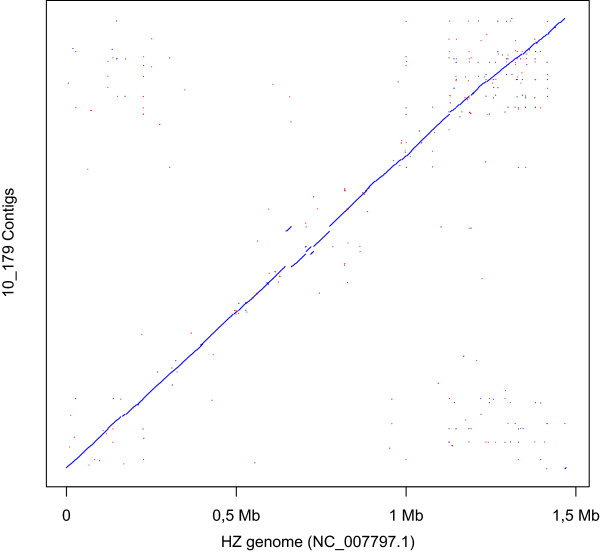
**Dot plot of assembled scaffolds versus HZ genome.**

**Figure 3:**
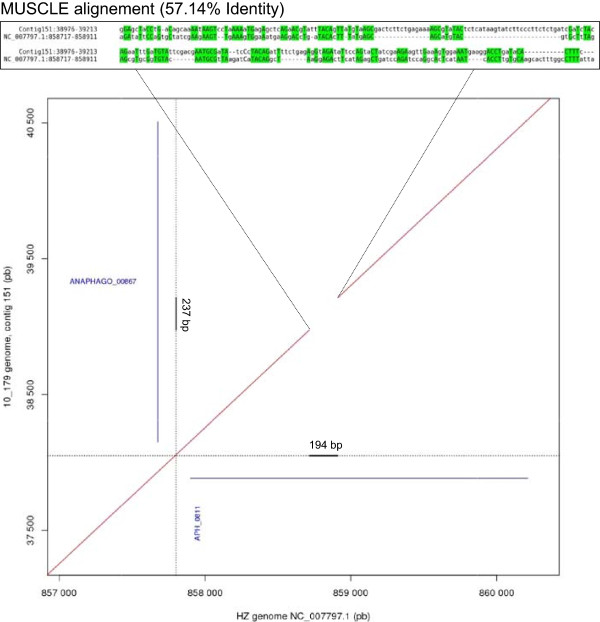
**Dot plot of contigs 151 versus HZ genome.**

After the assembly of the BOV-10_179 genome, 85.3% of the sequences included in the scaffolds aligned to the HZ genome (Figure 2). The 14.7% remaining sequences within the scaffolds did not map to the genome. These regions could either be absent from the HZ genome and/or too variable to correctly align with the HZ genome (Figure 3). On the other hand, only 79.2 % of the HZ genome sequence aligned to BOV-10_179 scaffolds. For the remaining HZ regions it was not possible to determine if they were indeed absent from the BOV-10_179 genome, or if they were missing due to biases introduced during the amplification steps (*i.e.* PCR) or because of biases related to the capture steps (*i.e.* hybridization). Using BOV 10_179, new capture probes could be designed, which should improve the capture rate of *A. phagocytophilum* DNA in bovine field samples.

In order to calculate bacterial DNA fold-enrichment, we estimated the amount of both *A. phagocytophilum* DNA mass and bovine DNA mass in sample 10_179 using six different qPCR amplifications: three which targeted *A. phagocytophilum* genes and three which targeted bovine genes (Table 1). The relative abundance of the *A. phagocytophilum* DNA within the original 10_179 sample corresponded to approximately 0.3% of the total DNA by mass (Table 2). Since the relative abundance was approximately 59% after the whole genome capture step, the level of enrichment was 197-fold, a rather high value. In fact, results obtained from other similar technical contexts appear to range from ~20-fold to ~100-fold [26, 29], and in some cases the level of variability among samples was significant [26]. The value obtained in this study could be explained by considering that in our protocol we used 300 μg of Cot-1 bovine DNA during the hybridization step, instead of 100 μg as suggested by the NimbleGen standard method. This modification could have increased the level of capture specificity by reducing non-specific hybridization.

**Table 1:** **Genes targeted and primers used for quantification of bovine and** ***A. phagocytophilum*** **DNA**

Organism	Gene targeted	Primer Sequences 5' - > 3'	Amplicon size (bp)	Reference
*A. phagocytophilum*	*ankA*	F: CTATCATCCTTGGGTAGTGGCCT	223	This study
		R: CTTCTGGTGTTCTCGGACCTTC		
	*gltA*	F: ATATCTACCGGAACCCCCATAGC	209	
		R: AAAAGCTATGACCCTAGAGCGCGT		
	*groEL*	F: GTGATTCGCACGCTCTTAGCAGT	172	
		R: GCCTTCAAGTTGCTGCTGTAAA		
*Bos taurus*	*gapdh*	F: TTCACACTCTCCTTCCAGGTACG	217	[30]
		R: TCAGGGCCTTAGAGATGGAAA		
	*ywhaz*	F: TACTCCGGACACAGGTAAGGGAC	212	
		R: AGAACCCAGCGGAAGAGAAG		
	*ppia*	F: CTGTTCTAGGTTGGATGGCAAGC	137	
		R: TTTGTCCACAGTCAGCAATGGTGA

**Table 2:** **Relative abundance of** ***A. phagocytophilum*** **DNA within the original 10_179 sample**

Gene targeted	Concentration of***A. phagocytophilum***DNA (μg/mL)	Average	Gene targeted	Concentration of bovine DNA (μg/mL)	Average	Relative abundance of***A. phagocytophilum***DNA (%)
*ankA*	0.34		GAPDH	101.905		
*groEL*	0.315	0.375	PPIA	53.18	129	0.3
*gltA*	0.475		YAHCR	232.395		

As previously stated, we obtained approximately 986X genome coverage, an extremely high value, much higher than that required to achieve a whole genome assembly of high quality (around 100X). Such extensive coverage was most likely generated during the experimental planning stage, as we were unable to predict the enrichment level achievable with an initial starting concentration of only 0.3% *A. phagocytophilum* DNA. Therefore, to ensure that the read number would be sufficient, we used an entire HiSeq 2000 lane for the sequencing. This first experience shows that it should be relatively simple to sequence several *A. phagocytophilum* genomes via multiplexing, without compromising the final results in terms of coverage. The only possible issue is that the enrichment ratio could in fact be rather variable from sample to sample, as observed by Bright *et al.* [26].

In conclusion, we have successfully demonstrated the effectiveness of a whole genome capture approach to selectively enrich for pathogenic bacterial DNA that was originally mixed with host mammalian DNA. This is the first report where an *A. phagocytophilum* genome sequence has been obtained without any culturing steps. As previously stated, whole genome capture has already been used to sequence the genome of the arthropod non-pathogenic symbiont *Wolbachia*, another obligate intracellular bacterium [5, 25]. Here, we demonstrate that whole genome capture is also a suitable approach for pathogenic bacteria WGS such as *A. phagocytophilum*, and that it could be adapted for large-scale studies of these bacterial genomes, directly from field samples.

### Features of *A. phagocytophilum*genomes

*A. phagocytophilum* has a single circular chromosome, without any identified plasmids [9]. The main characteristics of the ten genomes investigated in this study are summarized in Table 3. For BOV-10_179, the overall GC content was 41.6% as for other *A. phagocytophilum* genomes. This value is very high compared to other *Rickettsiales*-order bacteria, as the genome decay of these bacteria is often accompanied by a low GC% content [31–33]. A total of 1281 CDS (coding DNA sequences) were predicted, of which 1001 were complete. In addition, 3 rRNA and 37 tRNA coding sequences were also identified. Overall, genome size appeared to be conserved among the ten strains (approximately 1.5 Mb), whereas the number of genes varied greatly (from 1041 in the 10_179 genome, to 1411 in the HZ strain genome). These differences could be explained, at least in part, by fragmentation of BOV 10_179 incomplete sequence assemblies. Over 70% (717 of 1001) of the CDS had an assigned function.

**Table 3:** **Characteristics of the ten** ***A. phagocytophilum*** **genomes investigated in this study**

Strain	Host	Geographical origin	Genome size (Mb)	%GC	Number of genes*	Reference
HZ	Human (*Homo sapiens*)	USA	1.47	41.6%	1411	[9]
HZ2	Human (*Homo sapiens*)	USA	1.48	41.6%	1295	[10]
HGE1	Human (*Homo sapiens*)	USA	1.48	41.6%	1188	[10]
JM	Rodent (*Zapus hudsonius*)	USA	1.48	41.6%	1302	[10]
Dog2	Dog (*Canis lupus familiaris*)	USA	1.47	41.6%	1304	[10]
CRT38	Tick (*Ixodes scapularis*)	USA	1.51	41.6%	1202	[10]
CRT35	Tick (*Ixodes scapularis*)	USA	1.45	41.6%	1148	[10]
MRK	Horse (*Equus caballus*)	USA	1.48	41.6%	1155	[10]
10_179	Cow (*Bos taurus*)	France	1,37	41.5%	1041	this study
Norway variant2	Sheep (*Ovis aries*)	Norway	1.52	41.7%	1174	[10]

*genes include complete CDS, rRNA, tRNA and pseudogenes.

### Phylogenetic analysis

**Figure 4:**
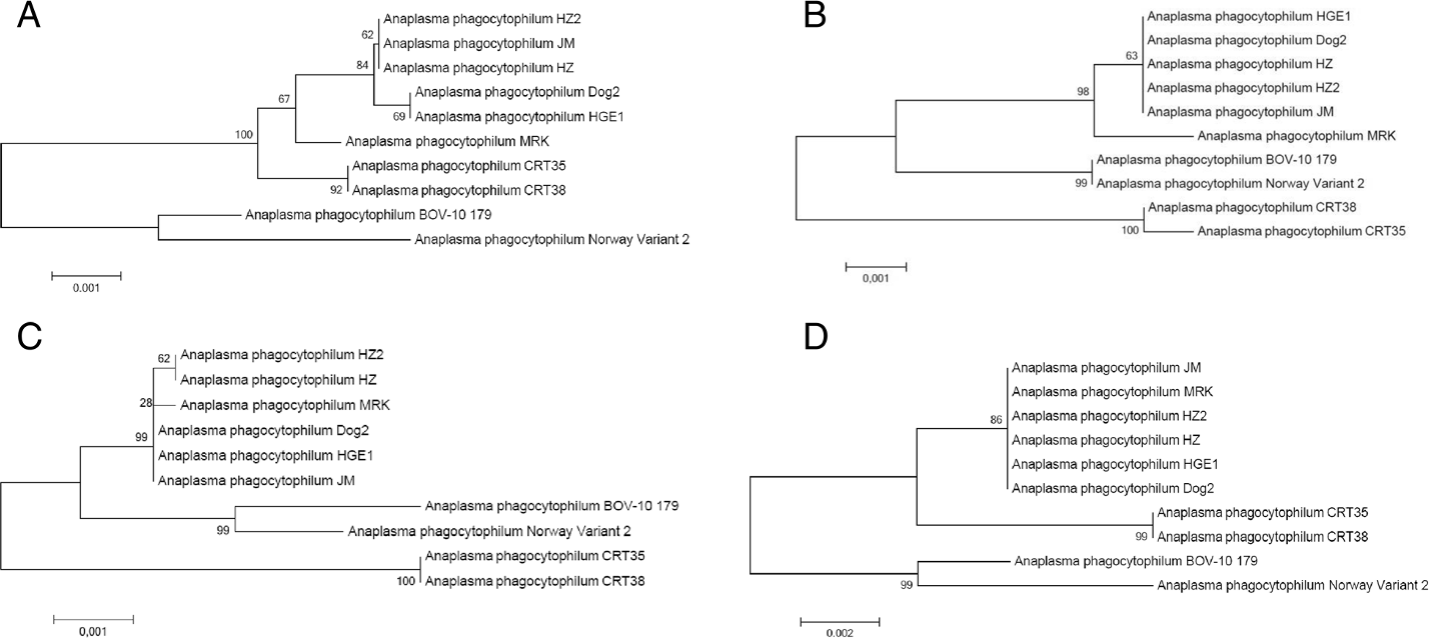
**Phylogenetic trees based on the** ***groEL*** **(A),** ***glta*** **(B), 16S rRNA (C) and** ***msp4*** **(D) loci.** Analyses were performed by the Neighbor-Joining method using 1000 bootstraps. The percentage of replicate trees in which the associated taxa clustered together in the bootstrap test (1000 replicates) are shown next to the branches.

Phylogenetic trees based on entire sequences of *groEL*, *glta*, *msp4* and 16S rRNA loci were constructed (Figure 4). The two European ruminant strains clustered together in all four phylogenetic trees. The CRT35 and CRT38 *Ixodes scapularis* strains were also associated within the four phylogenic trees, whereas the three human strains, the JM rodent strain and the Dog2 dog strain were always grouped together in the same separate cluster. Finally, the MRK horse strain clustered with the human strains only in the cases of 16S rRNA and *msp4* phylogenic trees.

### Comparison of the gene content of other *A. phagocytophilum*genomes

**Figure 5:**
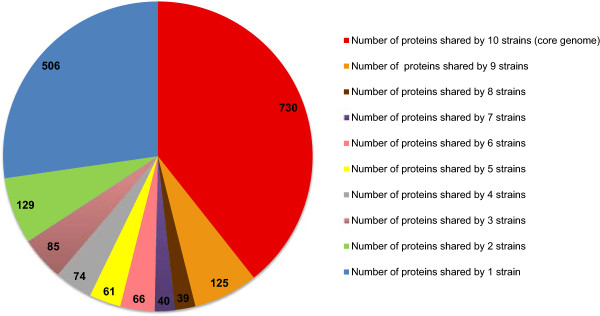
**Diagram of the number of proteins shared by different** ***A. phagocytophilum*** **strains.**

In order to compare gene content of the ten genomes investigated in this study, we first identified their core and accessory genomes by defining ortholog clusters at the protein level (see Methods). Following analysis, 1855 clusters were obtained, 730 (39.6%) of which belonged to the core genome (Figure 5 and Additional file 1). BOV-10_179 shared from between 770 to 795 proteins with the other nine strains considered in this study (Table 4). As for other *Anaplasmataceae*, in which the vast majority of genes belonged to the core genome [34–36], we were surprised to observe that *A. phagocytophilum* contained an exceedingly small proportion of such core genes. This could be due, at least in part, to the application of various annotation methods used for the ten studied genomes (for example, parameter setup and/or pseudogene definition), leading to differing gene predictions. In addition, it should be mentioned that (1) only the HZ, HZ2 and JM published genomes are fully sequenced (*i.e*. without gaps), and (2) the genome annotation of the HZ strain is of higher quality in comparison to the others, because proteomic data were also used [37]. Moreover, 506 clusters were found only in one strain (Figure 4). A large number of these clusters (210/506) corresponded to the *msp2*/*p44* gene family. This is not surprising, as these genes tend to have hypervariable central regions, therefore such low global similarity levels prevent their assembly into one cluster. Altogether, these factors could have led to a significant underestimation of genes belonging to the core genome. However, nine gene clusters were only detected in the genomes of two European domestic ruminant strains, and four additional clusters were unique to the genome from our bovine sample, when compared to available American *A. phagocytophilum* genomes (Table 5). As all corresponding proteins were annotated as "hypothetical proteins", their functions have yet to be explored. For instance, the proteins corresponding to the 13 aforementioned gene clusters could be involved in host tropism for those strains that infect domestic ruminants and/or could be specific to the European strains. In order to examine whether the differences between the European strains are related to animal species or to geographical location, the presence of these genes in *A. phagocytophilum* genomes from different wild and domestic animals living in the same areas should be investigated. The strategy developed in this paper would be an effective method to rapidly sequence these genomes.

**Table 4:** **Number of proteins shared between BOV-10_179 and the nine other strains considered in this study**

Strain	Number of proteins shared with BOV-10_179
HZ	781
HZ2	795
HGE1	784
JM	792
Dog2	793
CRT38	781
CRT35	770
MRK	774
Norway Variant 2	782

**Table 5:** **CDS specific to European ruminant strains, corresponding to hypothetical proteins of unknown function**

Present in	Locus tag
Cattle strain only	ANAPHAGO_00499
	ANAPHAGO_00588
	ANAPHAGO_00675
	ANAPHAGO_01075
Cattle and sheep strains	ANAPHAGO_00035
	ANAPHAGO_00070
	ANAPHAGO_00187
	ANAPHAGO_00232
	ANAPHAGO_00433
	ANAPHAGO_00566
	ANAPHAGO_00589
	ANAPHAGO_00885
	ANAPHAGO_01047

### Small peptides

Genes encoding small peptides (<100 amino acids) are common features of both prokaryote and eukaryote genomes, and are involved in many biological functions [38, 39]. For *Staphylococcus aureus*, one small peptide class is involved in both virulence and modulation of host immune responses [40].

In BOV-10_179, we detected 261 CDS (26.1% of the total predicted CDS) which coded for small peptides. All predicted small peptides had either no known function or were designated as p44/msp2 pseudogenes. In total, 31% (422/1357) of predicted CDS in the HZ genome coded for small peptides [9, 37]. Of these small peptide coding sequences, 67% (283/422) are actually produced [37], confirming that the majority are not only due to false positive gene prediction during genome annotation. However 82% (234/283) of the expressed small peptides have unknown functions [9, 37]. In order to better understand the biology of *A. phagocytophilum*, the functions of these small peptides must be explored.

### Adhesion and host cell internalization genes

Both the adhesion to and internalization of *A. phagocytophilum* within host cells are mediated by multiple bacterial adhesins/invasins that cooperatively recognize host cell receptors. To date, at least six *A. phagocytophilum* adhesins/invasins have been identified. These six proteins belong to the core genome of the ten strains assessed in this study. OmpA binds to α2,3-sialic acid of the sialyl Lewis x-tetrasaccharide that caps P-selectin glycoprotein ligand-1 (PSGL-1), and Msp2(p44) appears to recognize α-1,3-fucose and/or PSGL-1 N-terminal peptide [41]. Asp14 and AipA both recognize an unknown receptor [41, 42]. *A. phagocytophilum* adhesion could also involve Asp55 and Asp62, two other *A. phagocytophilum* surface proteins, as indicated by neutralization studies [43].

In BOV-10_179 and CRT38 strain genomes, we detected a 14-amino-acid-deletion in the N-terminal region of Asp14. The Asp14 domain essential for cellular adherence and invasion is located between the 12 to 24 C-terminal amino acids (amino acids 101 to 113) [44], therefore this deletion is not expected to have any effect on bacterial adhesion. Interestingly, we detected one substitution at position 118 (alanine - > threonine) which was only observed in ruminant strains.

The OmpA protein is completely conserved between strains, except for the domestic ruminant variants; Norway Variant 2 and 10_179, in whom five substitutions were detected. However, as these substitutions lie outside the essential binding domain of OmpA [45], they are also not expected to have any effect on bacterial adhesion.

In the HZ strain, AipA is a 355 amino acid protein, however in CRT35, Dog2, JM, MRK and HZ2 strains, we detected a deletion of 118 amino acids in the AipA N-terminal region. In strains 10_179, CRT38 and HGE1, we detected an additional deletion of four amino acids in the same N-terminal region. However a nucleotide BLAST search of the HZ *aipA* nucleic sequence revealed that this gene is actually intact in the ten studied genomes. The start codon for this gene is “TTG, therefore this inaccuracy could be due to difficulties with start codon detection during genome annotation. We also detected 49 deletions and substitutions in AipA proteins across the ten strains. Further analyses are required in order to draw conclusions on the functional impact of these variations.

The HZ genome contains one *msp2* locus, two *msp2* homolog loci, and 113 *p44* loci [9]. In order to evade the host immune system, *A. phagocytophilum* utilizes gene conversion to shuffle *msp2* pseudogenes into the single *msp2* gene expression cassette [46–50]. One proteomic study has shown that in addition to the expression site APH_1221, full-length *p44* genes can be expressed at their own loci, whereas silent *p44* genes have to be recombined into the expression site for protein production [37]. The locus (or loci) expressed during the interaction of Msp2 with PSGL-1 is/are not known. Thus it was not possible to further investigate the role of msp2/p44 variations in *A. phagocytophilum* binding. Moreover the binding sequence on Msp2 remains undefined.

Asp55 and Asp62 are the least conserved proteins across all the strains, containing 58/558, and 99/583 amino acid substitutions or deletions, respectively. However protein characterization studies must still be performed to determine the functional impact of these variations.

### Genes encoding proteins involved in secretion systems

Protein export systems are extremely important for host-pathogen interactions. Gram-negative bacteria often contain the general secretion (Sec) and the twin-arginine translocation (Tat) pathways, both involved in exporting protein into the periplasm; and specialized export systems, such as the type IV secretion system, dedicated to exporting specific subsets of proteins (reviewed in [51]). All ten strains studied in this work contained both the Sec and Tat pathways, as well as type I and type IV specialized export systems.

#### Sec pathway

All strains contained eight genes (*secA*, *secB*, *secD*, *secE*, *secF*, *secG*, *secY*, and *yajC*) of the Sec pathway (for a review see [52]).

Detection of potential Sec substrates was performed as described in the material and methods section, and are listed in Additional file 2. Many proteins detected as potential Sec substrates are surface outer membrane proteins (Asp55, Msp2, Msp4, OmpA, Omp85, Omp1X, NlpD, APH_0625, APH_1110). These proteins may be secreted into the bacterial periplasm by the Sec pathway, and then anchored in the outer membrane.

The VirB2, VirB6 and VirB9 subunits of the type IV secretion system have also been detected as potential Sec substrates. VirB2 and VirB9 proteins are periplasmic and/or outer membrane channel subunits of the T4SS, and VirB6 could be surface exposed in *A. phagocytophilum* [53, 54]. The Sec pathway may secrete these proteins into the bacterial periplasm for assembling, whereas other Vir proteins are localized to the inner membrane of *A. phagocytophilum*, and do not require secretion.

Seven proteins (APH_0208, APH_0232, APH_0561, APH_0687, APH_0957, APH_1084, and APH_1148) involved in various cellular processes such as cell metabolism, and two lipoproteins (APH_0985, APH_1087) with unknown localization, were also detected as potential Sec substrates.

Ten hypothetical proteins of unknown function were also identified. These proteins could be uncharacterized outer membrane proteins, or might perform their biological activity in periplasmic compartments; consequently their functions need to be explored.

The majority of the proteins considered as potential Sec substrates were detected in all ten genomes. However some of these proteins were only detected in some of the compared genomes, such as CRT38_03582 which was only present in the CRT38 and CRT35 genomes (Additional file 2).

#### Tat pathway

The Tat pathway is found in most bacteria and has been proven to be essential for virulence in several pathogens [55]. In *Escherichia coli and Anaplasma marginale*, the minimal set of genes required for Tat translocation and a functioning Tat system consists of *tatA*, *tatB*, and *tatC* [56, 57]. All strains examined in this study contained *tatA*, *tatB*, and *tatC*, suggesting that they produce functional Tat pathways. It is interesting to note that while Tat proteins are not synthesized during HL-60 cell infection [37], *tatA* is expressed in tick salivary glands, suggesting that *A. phagocytophilum* may utilize the Tat pathway during tick infection [58]. Contrary to many other α-proteobacteria, but consistent across *Rickettsiae*, the three *tat* genes are dispersed throughout the genome [57].

Our *in silico* investigations led to the prediction of two potential Tat substrates across the ten *A. phagocytophilum* strains. The first had 33% and 28% amino acid identity with the OmlA protein of *Azospirillum sp* and *Wolbachia* wNo, respectively. OmlA is an outer membrane lipoprotein involved in the maintenance of cell envelop integrity in *Pseudomonas aeruginosa* [59], and possibly virulence in *Xanthomonas axonopodis* [60]. As lipoproteins are particularly immunogenic and often have adjuvant activity, OmlA could be a useful vaccine candidate for pathogenic bacteria. As such, it has already been used in pigs to protect against *Actinobacillus pleuropneumoniae* [61]. The fact that OmlA is present in all the *A. phagocytophilum* strains studied here, reinforces its potential efficacy as a vaccine candidate against this bacterium. However, this protein is not conserved across all strains. For example, a deletion of approximately 60 amino acids, containing a cysteine residue involved in lipid attachment, is observed in the OmlA N-terminal region of the HZ strain. This observation suggests that OmlA may not be a surface-exposed protein in the HZ strain, raising doubts about the utility of this protein as a vaccine candidate. The second potential Tat substrate is a ubiquinol-cytochrome c reductase iron-sulfur subunit involved in the electron transport chain.

To date, both the Tat and Sec pathways have been poorly studied in *A. phagocytophilum.* Due to their importance in other pathogenic bacteria, further investigations are needed in order to fully explore their specific roles in *A. phagocytophilum* virulence.

#### T1SS secretion system

The type I secretion system (T1SS) spans the periplasm and both inner and outer membranes of Gram-negative bacteria, and facilitates protein secretion across these compartments. The T1SS is composed of three major proteins: ATP-binding cassette (ABC) transporters, outer membrane factors (OMF), and membrane fusion proteins (MFP) [51]. All ten strains in this study contained these three protein-encoding genes. It remains to be elucidated whether these genes code for an efficient T1SS. However, the strong conservation of the genes encoding this secretion system that has evolved through reductive evolution, observed across all ten *A. phagocytophilum* genomes, [33], suggests its importance in the bacterial lifecycle. Many T1SS substrates are involved in bacterial virulence (for a review see [62]). In *Ehrlichia chaffeensis*, three secreted proteins containing tandem repeats and the ankyrin repeat protein, Ank200, involved in host pathogen interactions, are T1SS substrates [63]. To date, no T1SS substrate has been described in *Anaplasma spp*. Identifying TSS1 substrates may be a significant step towards a better understanding of *A. phagocytophilum* biology.

#### T4SS secretion system

The type IV secretion system (T4SS) is a multi-protein complex that also spans the periplasm and both membranes of Gram-negative bacteria. It can also span eukaryotic host cell membranes and has the ability to transport both nucleic acids and proteins into eukaryotic host cells, and to interfere with host signaling. The T4SS is essential for the survival and virulence of many intracellular bacteria [64], and the structure of the *A. phagocytophilum* T4SS has been well studied [9, 53, 65]. Each strain studied here contained one copy of *virB3*, *virB10*, *virB11*, and *virD4*; two copies of *virB4* (one copy of which was truncated in all strains, whereas the Norway Variant 2 carried two truncated copies); *virB8* and *virB9*; four copies of *virB6* (*virB6-4* was incomplete in CRT38, HGE1, and 10_179 strains likely because it was located near contig termini); and a variable number of *virB2* homologue genes. VirB2 proteins constitute the secretion channel and are the most diverse of the T4SS subunits in *A. phagocytophilum*, both in terms of copy number and sequence [53]. We found 7 homologue *virB2* genes in the HGE1 genome, 8 in HZ, HZ2, and 10_179 genomes, 9 in Dog2, JM, MRK, and Norway Variant 2 genomes, 13 in the CRT35 genome, and 15 in the CRT38 genomes. As already described in previous studies, we found that all strains analyzed here lacked both *virB1* and *virB5* genes [9, 53].

The role of T4SS in *A. phagocytophilum* virulence has been well studied. Two *A. phagocytophilum* T4SS substrates are currently recognized: i) the ankyrin repeat domain-containing protein A (AnkA), which interacts with host proteins and DNA [66–68], ii) the *Anaplasma* translocated substrate 1 (Ats-1), involved in the induction of autophagosome formation and potential interference with apoptosis induction [69–71]. The *ats-1* gene was detected in all ten genomes in this study, whereas *ankA* was absent from the CRT38 genome. As the CRT38 genome was composed of two contigs, separated by a gap, we cannot conclude if *anKA* was really absent from this genome, or if the gene was not sequenced or assembled because it happened to fall within the gap.

#### Other secretion systems

Genes representing components of other secretion systems (types II, III, V, VI) were not detected in any of the genomes analyzed here, as previously described for the HZ genome [9]. However, some proteins, such as APH_1235, which lack a secretion signal, have been detected on the bacterial surface, indicating that *A. phagocytophilum* may have alternative motifs directing proteins to cell surface [58].

## Conclusion

In this study we have used an innovative approach in order to sequence the genome of a bovine sample of *A. phagocytophilum*, and compare it to other published genomes.

Whole genome capture has already been successfully used for *Wolbachia* genome studies [5, 25]. Here we demonstrated that this approach is also applicable to a pathogenic bacterium, *A. phagocytophilum,* and permits the sequencing of whole genomes without any need for strain isolation. For this reason, we strongly believe that our approach generates a promising tool for large-scale studies of *A. phagocytophilum* genomes, directly from field samples. Genome comparison allowed us to identify four proteins specific to the *A. phagocytophilum* bovine genome, and nine proteins specific to the two available European domestic ruminant strains. As these proteins could be involved in ruminant strain host tropism, their functions necessitate further exploration.

## Methods

### Ethics statement

The domestic animals used in this study met the definition of “farm animals”, which are not covered by French regulations (Decree n° 2013–118 implemented the 1^st^ February 2013 issued by the French Ministry of Agriculture). The animal owners provided permission for studies on samples issuing from their animals.

### Samples collection and diagnosis of *A. phagocytophilum*infection

#### Sample collection

Blood samples were collected from cow 10_179 suspected of having TBF (originating from Grazac, Tarn French Department, in the southwest of France), and from bovine 1, which did not show any apparent clinical signs of TBF. The *A. phagocytophilum* genome sequence obtained from cow 10_179 is designated as BOV-10_179.

#### Morula examination

Blood smears were used to visualize *A. phagocytophilum* morulas in neutrophils. Smears were prepared using 200 μL of blood, stained using the May-Grünwald Giemsa stain (CML, Nemours, France), and then observed under a microscope. A sample was considered as negative for TBE infection if no morulas were detected across the entire surface of a blood smear.

#### *msp2*PCR amplification

*msp2* (major surface protein 2) PCR amplification was performed as previously described [72].

### DNA extraction and dilution

DNA was extracted from the purified *A. phagocytophilum* Webster strain, and from bovine 1 and cow 10_179 blood samples, using the NucleoSpin® Blood QuickPure kit (Macherey-Nagel, Bethlehem, USA) according to manufacturer’s instructions. DNA concentrations were determined using the NanoDrop 2000 spectrophotometer (Thermo Fisher Scientific, Waltham, USA). DNA dilutions ranging from 1 to 10^-4^ were prepared by 10-fold serial dilution. DNA samples were stored at -20°C prior to use.

### qPCR quantification of bovine and *A. phagocytophilum*DNA proportions

In order to assess the proportions of *A. phagocytophilum* DNA mass *versus* bovine DNA mass in sample 10_179, six different qPCR amplifications were performed: three targeted *A. phagocytophilum* genes and three targeted bovine genes. The targeted genes, and the corresponding primers used for each test are described in Table 1. Serial DNA dilutions from the Webster strain and bovine 1 were used to create standard curves for each targeted gene. qPCR assays were performed using the Maxima SYBR Green qPCR Master Mix (2X) Kit (Thermo Fisher Scientific) in a 25 μl total reaction volume, with Master Mix at a 1X final concentration, 0.3 μM of each primer and 5 μl of purified DNA. Negative controls were included in each run. qPCR cycling was performed on the LightCycler480 Multi-well Plate 96 system (Roche, Basel, Switzerland) as follows: 95°C for 10 min, then 40 cycles of 10 s at 95°C, 30 sec at 60°C and 30 s at 72°C. The signal emitted was detected at the end of each annealing-extension step. A threshold was automatically set and the threshold cycle value (Ct) was consequently determined. Two replicates of the assay within and between runs were performed. Concentrations of *A. phagocytophilum* and bovine DNA in sample 10_179 were calculated by comparing the Ct of the sample to the standard curves.

### Capture array design

To design the capture array, the *A. phagocytophilum* sequence was downloaded from NCBI (accession NC_007797.1). The genome was 1,471,282 bp in length. The custom Sequence Capture 2.1 M array was designed by Roche-NimbleGen (Madison, USA) using standard parameters, except for the probe unicity constraint which was removed, allowing each probe to match the reference up to 25 times.

### Library preparation

The NimbleGen solid phase capture protocol required 5 μg of PCR-amplified genomic library DNA, and since the typical amount obtained from a single reaction ranges from 0.75 to 1.25 μg, five libraries were prepared using the same *A. phagocytophilum* genomic DNA and subsequently pooled for the hybridization step.

For each library, 1.5 μg of gDNA were measured using the Qubit apparatus (Invitrogen, Carlsbad, USA). DNA was then resuspended in 130 μL of ddH2O and fragmented with a Covaris S-2 instrument (Covaris, Woburn, USA) using the following settings: number of cycles: 6, duty: 5%, intensity: 4, cycles/burst: 100, duration: 210 sec. Randomly sheared-DNA was purified using 1.8X AMPure beads (Beckman Coulter Genomics, Danvers, USA) and resuspended in a final volume of 60 μL Resuspension Buffer (Illumina, San Diego, USA). For each sample, 1 μL was then run onto a DNA 1000 Bioanalyzer chip (Agilent Technologies, Santa Clara, USA) for quality control.

For all downstream steps of end-repair, A-tailing, and adaptor ligation, the TruSeq DNA Sample Preparation kit (Illumina) was used following manufacturer’s recommendations. The only departure from the protocol concerned the step of agarose gel size selection, which was skipped. All libraries were produced using Index 1.

After ligation, indexed samples were PCR-amplified (‘pre-capture enrichment’) and their quality checked by quantifying with a Qubit and using DNA 1000 Bioanalyzer chips.

The hybridization step was performed using a single 385 K array (Roche-NimbleGen), and the hybridization cocktail was prepared as follows. First, five genomic libraries were pooled to obtain a final amount of 5 μg of DNA. Subsequently, 300 μg of *B. taurus cot-1* DNA (Applied Genetics Laboratories, Melbourne, USA) and 10 μL of each of the two blocking oligonucleotides ‘TS-HE_Oligo_1’ and ‘TS-HE_Index_1_Oligo’ at 100 μM (Eurofins MWG Operon, Ebersberg, Germany) were added.

The cocktail was then dried in a SpeedVac at 60°C. For the downstream steps of hybridization, washing, and elution the protocol suggested by NimbleGen (NimbleGen Array User’s Guide, Version 3.2) was used.

Afterwards, the eluted library was again amplified via PCR (‘post-capture enrichment’). The protocol suggested by the manufacturer (Illumina) was adopted, with 12 PCR cycles. Eventually, the library was quantified using a Qubit, and run onto DNA 1000 Bioanalyzer chips.

### Sequencing, genome assembly and annotation

The sequencing of the captured library was performed on an entire lane of a flow cell on a HiSeq2000 (Illumina) sequencer as paired-end 108-bp reads. Base calls were performed using RTA software.

After removing low quality bases (PHRED < 10) from 3’ read extremities, the reads were mapped to the genomes of *B. taurus* (UMD3.1) and *A. phagocytophilum* HZ (NC_007797.1) using bwa (v 0.6.1) [73] with default parameters.

After this step, the reads mapping to the *B. taurus* genome where filtered out. Therefore, only the properly paired reads mapping to the *A. phagocytophilum* genome and the properly paired reads which where unmapped on both genomes, were retained. PCR duplicates were detected on the subset of reads which were mapped to the pathogen genome and then subtracted using samtools rmdup [74]. On the other hand, the filtering of duplicated reads was not carried out on unmapped reads.

The *A. phagocytophilum* properly paired reads retained after the removal of duplicates were combined to the properly paired unmapped reads to obtain a final dataset.

Before the assembly step, the reads were digitally normalized using Khmer [27, 28] with a kmer size of 20, and a cutoff of 20. 17% of paired-reads were conserved.

Remaining reads were then assembled using Velvet 1.2.07 [75], with a kmer value between 51 and 91, and default parameters.

Genome annotation was performed using Agmial [76]. Functional annotation was performed by preferentially transferring annotations from the *A. phagocytophilum* HZ strain where appropriate.

The 169 scaffolds have been deposited in the European Nucleotide Archive (CCXQ01000001-CCXQ01000169).

### Genome alignment

The 169 assembled scaffolds were aligned against *A. phagocytophilum* strain HZ (NC_007797.1) using MUMmer 3.0 software [77]. All perfectly matching regions were determined using the mummer module with default parameters, and were used to determine coverage statistics. Then the Nucmer module was used with default parameters to cluster and extend exact matches in order to create the largest similarity regions. These large regions were then used to generate dot-plots.

### Core genome determination

The BOV 10_179 genome sequenced in this study (CCXQ01000001-CCXQ01000169), and the genomes of the previously sequenced Human strains HZ (NC_007797.1), HZ2 (NC_021879.1), HGE1 (APHH01000001.1 and APHH01000002.1), the dog strain Dog 2 (NC_021881.1), the horse strain MRK (PRJNA216999), the rodent (*Zapus hudsonius*) strain JM (NC_021880.1), the sheep Norway Variant 2 (PRJNA217033) and the *Ixodes scapularis,* collected from white-tailed deer, strains CRT35 (PRJNA217037) and CRT38 (APHI01000001.2 and APHI01000002.1) were compared for core genome determination.

Complete protein sequences encoded by non-pseudogenes in the ten different genomes were compared using protein BLAST to define groups of orthologous proteins by single linkage clustering (e-value <10-3; >70 % identity over >98 % of the longest sequence length).

### Phylogenetic analysis

Nucleotide sequences of *groEL*, *glta*, *msp4* and 16S RNA loci were downloaded from GenBank, and analyzed using the program MEGA6 (Molecular Evolutionary Genetics Analysis Version 6.0) [78]. Sequences of each gene were aligned by ClustalW applying the IUB matrix. Alignments have been deposited in TreeBase (http://treebase.org/treebaseweb/home.html). Tree construction was achieved using the Neighbor-Joining method [79]. The percentage of replicate trees in which the associated taxa clustered together in the bootstrap test (1000 replicates) are shown next to the branches [80].

### *In silico*prediction of Tat and Sec substrates

Potential Tat substrates were detected as described previously [57].

Within the protein set coded by the annotated genomes of *A. phagocytophilum*, available at NCBI, type I signal peptides and their cleavage positions, corresponding to sequences specific to potential Sec substrates, were sought using three existing programs for sec signal prediction: SignalP 4.1 (http://cbs.dtu.dk/services/SignalP//; [81]), Signal-3 L (http://www.csbio.sjtu.edu.cn/bioinf/Signal-3L/; [82]) and Signal-BLAST (http://sigpep.services.came.sbg.ac.at/signalblast.html [83]). SignalP incorporates cleavage site prediction and signal peptide/non-signal peptide prediction, based on a combination of several artificial neural networks and hidden Markov models. Signal-3 L is an automated method for predicting signal peptide sequences and their cleavage sites in protein sequences. It consists of three prediction engines interpreting three progressively complex layers: i/ identifying a query protein as secretory or non-secretory via an ensemble classifier; ii/ using a sub-site-coupled discrimination algorithm to select candidates for possible signal peptide cleavage sites in a query secretory protein; iii/ determining the final cleavage site by fusing the global sequence alignment outcome for each of the aforementioned candidates through a ranking system. The Signal-BLAST algorithm performs signal peptide prediction based on sequence alignment techniques. All programs were run from their respective servers. We considered that a protein contained a putative Sec signal if it was predicted by at least two of the software programs.

### Multiple protein alignments

Proteins multiple alignments were performed using MUSCLE (MUltiple Sequence Comparison by Log Expectation) software, in MEGA6 environment [78]. The parameters used were: gap opening penalty: 10; and gap extend penalty: 0.1.

### Availability of supporting data

All the supporting data are included as additional files.

## Electronic supplementary material

Additional file 1: **Core and accessory genes of ten** ***A. phagocytophilum*** **genomes.** (XLSX 366 KB)

Additional file 2: **Potential Sec substrates detected in ten** ***A. phagocytophilum*** **genomes.** (XLSX 10 KB)

## Abbreviations

ABC: ATP-binding cassette
ankA: ankyrin repeat domain-containing protein A
Ats-1: Anaplasma translocated substrate 1
BLAST: Basic Local Alignment Search Tool
CDS: Coding DNA sequences
HGA: Human granulocytic anaplasmosis
MFP: Membrane fusion proteins
MEGA: Molecular Evolutionary Genetics Analysis Version
MUSCLE: MUltiple Sequence Comparison by Log Expectation
NGS: Next-generation sequencing
OMF: Outer membrane factors
PSGL-1: P-selectin glycoprotein ligand-1
T1SS: Type I secretion system
T4SS: Type IV secretion system
Tat: Twin-arginine translocation
TBF: Tick-borne fever
WGS: Whole genome sequencing.
